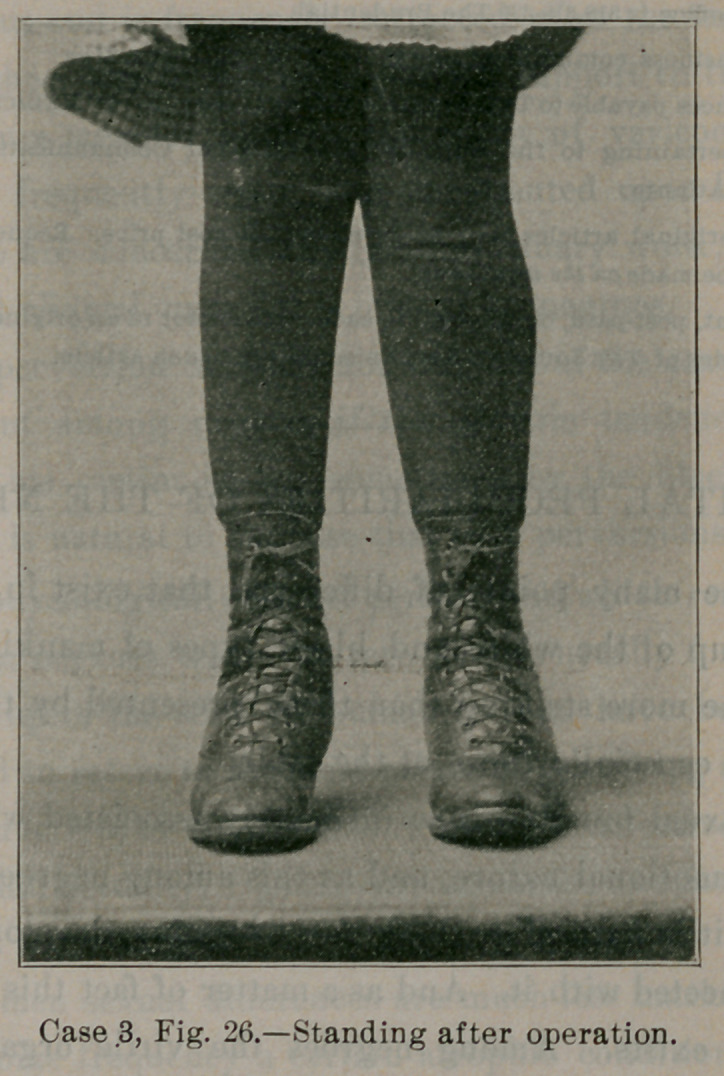# A Brief Study of Flat-Foot

**Published:** 1903-03

**Authors:** Michael Hoke

**Affiliations:** Orthopedic Surgeon to the Presbyterian Hospital, Atlanta, Ga.


### A BRIEF STUDY OF FLAT-FOOT.

BY MICHAEL HOKE, M.D.,
Orthopedic Surgeon to the Presbyterian Hospital, Atlanta, Ga.

This paper is based on observations made upon twenty-seven flat-foot and fifty-two pronated foot cases. A study showing the relative frequency of their common and somewhat rare features would be tedious. Hence, rather than tabulate a mass of varying detail, I have preferred to embody in a general consideration of the subject the main simple, useful ideas these cases suggest.

The clearest conception of the human foot's function, the preservation of that function, and its restoration when it has been impaired or lost, can be had by looking upon the foot as a lever, and as an anatomical bridge. It has its arches composed of bones, bound together by ligaments. As a lever, the power is applied at the posterior end, the heel, and behind the ankle; the fulcrum is at the toes; the weight is applied at the ankle-joint, between these two. The calf muscles, whose bellies lie above, by the familiar attachment of their tendons to these arches, may make them rigid or permit a flexible rise and fall under the influence of the body weight, or may move a part or the whole of the structure as a unit. In addition to sustaining the body weight it has to be the instrument by which the body is propelled. This should be easy, painless, graceful. Thus poise produced by the coordinate action of the muscular supports, each muscle responding proportionately to a weight stimulus, is necessary for the organ's activity as a unit.

For these structures to do their work properly too great a strain must not be applied to one segment of the bridge; nor one, nor a group of muscles taxed more than is just, considering their size, anatomical positions, and the mechanical conditions under which they work. If a group of muscles is overstrained, that part of the arch they strengthen, and whose action they control, begins to yield, and the ligaments stretch; the bones rotate to unnatural limits; pain and disability follow. If the process is stopped at this point, a slight static defect results; if the organ is used too much or improperly in this partly crippled condition, the tarsal bones rotate further; there is more permanent stretching of ligaments. If this continues, in addition to the changed position of the bones, the changed mutual pressure of adjacent bones produces here body absorption; there exostoses, and deforms the skeleton.

The human foot follows the simple laws obeyed by similar structures sustaining weight. A study of flat-foot resolves itself then into an inquiry into how the foot best carries its burden, what is the best distribution of the weight it bears, and, once a structural weakness has been produced, how one may best restore the organ's usefulness.

Presuming the foot to be intact, that the bones have the normal relationship to one another, that the muscles are strong, that there be power in the toes, that the various ranges of motion are possible to the natural limits, it is advantageous to use it in a certain manner. Observations upon the gait of athletes, agile savages, the art of the ancients, upon all the best types of the physical man, show that in walking and running the foot is placed so that its long axis points almost directly in front. When this is done the inner edge of the great toe, the inner malleolus and the inner condyle of the femur are in the same plane, and the line of the crest of the tibia, if prolonged downwards, passes off the dorsum of the foot between the second and third toes. This position produces the greatest degree of strength, agility and poise, with the minimum of fatigue. Fig. 1, right leg, illustrates this position. Let this be called the strong position.

Fig. 1.

Fig. 2.—Imprint of normal foot on blackened paper.

With the foot in this position relative to the leg, the major portion of the body weight falls upon the external arch. This is illustrated by the imprint of the foot upon blackened paper, and by the casts taken in this position. See in Fig. 2, imprint of a normal foot, that the areas of pressure are beneath the heel, the external arch, and the ball of the foot; the internal arch has not touched except at its two ends, the heel and the head of the first metatarsal bone. A view of the normal foot from the inside illustrates the same thing, shows a high arch beneath the internal row of bones; beneath the internal arch the soft tissues touch the surface.

For anatomical reasons this is as it should be. The external arch is very strong, resting almost flat upon the ground; it has only one joint, permitting little motion at that point; there is the strong calcaneo-cuboid ligament beneath holding the bones tightly together, put there for passive weight-bearing; there are no muscular attachments to this arch, hence the application of strain has little chance to produce fatigue. In this position [Fig. 1], the astragalus is tilted a little outwards, and its under surface rests directly upon the os calsis, so that comparatively little weight is transferred to the internal long arch by the head of the astragulus.

Fig. 3.—Rt. foot, inner longitudinal arch, W—Weight of body. X and Y—Components of body weight falling on inner arch tending to pronate the foot; Y is resisted by tendons (arrow) passing beneath sustentaculum tali, E; Y produces valgus rotation of the os calsis; X, the abduction of the front part of the foot, etc.

Fig. 4.

The internal long arches present the weakest parts of the foot; the weakest point in these arches is at the astragalo-scaphoid articulation, the inner end of the mediotarsal joint. This is necessarily so. The arch is very flexible, the number of joints in it permitting much motion, and it depends largely upon muscles for its support; these are in turn subject to fatigue.

The inner row of bones forms two arches, A, B, C, D, E, F, Fig. 3, A′, B′, C′, D′, E′, F′, Fig. 4. That part of the body weight which the internal arch bears, sufficient normally to cause a slight descent at D and inward excursion at D′, the astragalo-scaphoid articulation, giving elasticity to the step, is applied by the head of the astragalus at D and D′, the weakest points in these two arches. This is the inner end of the mediotarsal point, which may be termed the hinge, dividing the front part of the foot from the back part. Notice in Fig. 3 that the line of action of body weight applied to this arch is in direction of the axis of the astragalus, and if prolonged forward, passes through the head of the first metatarsal. Fig. 5 X-ray of normal foot in strong position, demonstrates this.

Fig. 5.—Skiagraph of normal foot.

So long as the larger portion of the body-weight strain falls upon the external arch, it is easy for the two internal arches to preserve their formation.

The tendency of the astragalus is to slip downward, forward and inward, the way its head points.

Fig. 6.

Arch A, B, C, D, E, F, Fig. 3, is increased by flexion of its interior end, the head of the great metatarsal. Conversely its tendency is to flatten if its anterior end is not kept flexed. The flexors of the great and small toes accomplish this when brought into active service. The action of the anterior tibial is to lift up the arch in its middle. If the anterior tibial is weakened the arch loses this support. It is seen how the head of the astragalus is kept from slipping downward, forward and inward.

Arch A′, B′, C′, D′, E′, F′, Fig. 4, is increased by adduction of its anterior end, the head of the great metatarsal, and is conversely weakened by abduction of its anterior end. The adductors of the great toe and tibialis posticus strengthen this arch when brought into play.

Fig. 7.—Skiagraph of pronated foot, early stage of flat-foot.

The tibialis posticus acts by pulling the scaphoid against the head of the astragalus, so as to check the discursions downward and inward of the latter.

It is manifest then that flexion and adduction of the front part of the foot is the best possible position for the preservation of the internal long arches. When the foot is used in this way, the body weight is distributed in a considerate manner, the outer arch bearing the greater part of the foot strain, the inner arch being reserved for that resiliency which, when present, produces an active and graceful form of locomotion. This advantageous posture of the foot is seen in the position of the right foot (Fig. 1).

In the normal foot these internal arches are held intact by the ligaments and the muscles, in the above described way.

Fig. 8.—Skiagraph of bad flat-foot. For description see remarks on Pathology.

Fig. 9.—Inprint of pronated foot.

Suppose, however, the body weight is differently distributed, so that the weak inner arch takes most of the strain, as illustrated in Fig. 6, in which case the line of the crest of the tibia passes off the foot at some point off the internal arch. Soon the muscles tire; they no longer preserve adduction and flexion; then yielding of the internal arches begins. In the early stages of flat-foot, that stage being illustrated by Fig. 6, a type of static defect called the pronated foot, arch A, B, C, D, E, F, is somewhat flattened, the line of the action of the body weight—*i. e.*, the prolongation of the axis of the astragalus—falls some distance inside of A′ (see Fig. 7). In a well-marked case of flat-foot A, B, C, D, E, F is flattened. Arch A′, B′, C′, D′, E′, F′ is reversed (see Fig. 8). The etiology of flat-foot then resolves itself into this: It is due to too much strain applied upon the inner long arches. That the strain in flat-foot is upon the internal arch is seen in figures 9, 10 and 11, imprints upon the blackened paper of a mild, ordinary and severe type of flat foot. Fig. 11 is the imprint of the same foot of which 8 is the skiagraph. Compare them with Fig. 2, impression of normal foot, and notice the thickness of the imprint in each stage, extending progressively further inward beneath the long internal arches, demonstrating that the more weight the internal arch bears the flatter it becomes.

Fig. 10.—Imprint of flat-foot.

Fig. 11.—Imprint of bad flat-foot.

The incidental factors producing these primal causes are:

(1) Using the foot with the toes turned out. Most women, because they are naturally knock-kneed, walk with the feet markedly turned out; combining this factor with false pride about shoes, and the fact that nine out of ten lead indolent lives, it becomes readily apparent why so many of them have pronated feet with consequent pain and physical disability.

(2) Improperly fitting shoes. If the toes are cramped, it is painful to use the flexors of the toes, painful to complete the step by rising on the toes, impossible to walk without pain except by abducting the front part of the foot; then if the forepart of the foot is not used, all the muscular work has to be done by the gastrocnemius and the tibial muscles. The latter imposed upon in this manner, soon tire out; then the ligaments alone stand the strain, they stretch; the astragalus slips downward, forward and inward; unless the process is checked, flat-foot results.

(3) Deflection of the tibia above the ankle, and bowlegs may change the line of action of the body-weight, so that the internal arches bear more than their proportionate part of the strain. They give way with the production of flat-foot.

(4) Flat-foot often follows rheumatism, and rheumatoid arthritis of the ankle and tarsus. Frequently the pain in the foot after an acute or long attack of these diseases is due not to the continuation of the disease, but to the changed condition of the tarsal bones and ligaments.

(5) Acute and chronic inflammation of the knees, followed by outward deflection of the leg, may produce the condition.

(6) Acute anterior poliomyelitis paralyzing one or more muscles, supporting the internal arch, produces flat-foot.

(7) Flat-foot may be congenital.

(8) Anemia, acute diseases, great body-weight, etc., manifestly cause weakness of the muscles.

(9) Improper walking during convalescence after bad sprains predisposes one to flat-foot.

The *pathological findings* are clearly what they ought to be from the above consideration of the dynamics of the condition. In acute cases and in mild cases, the so-called pronated foot, there is no change in the structure of the skeleton. The deformation is purely postural in the early stage, consists in abduction of the part of the foot in front of the mediotarsal joint, valgus rotation of the os calsis and a sliding downwards and inwards of the head of the astragalus, so that its longitudinal axis, if prolonged (see Fig. 7), passes a little distance internal to the head of the great metatarsal bone. If this continues, as time goes on, the ligaments attached to the inner part of the foot, particularly the inferior calcaneo-scaphoid and the calcaneo-astragaloid, become stretched by the pressure of the astragalus, the internal arches lose the mechanical accuracy of their form, the tibial group of muscles atrophy and their tendons stretch. The bones in their new abnormal posture are subjected to abnormal pressure, weight, ligament and tendon-tugging conditions, some parts of the skeleton atrophy, it grows in others which should be subordinate in size, thus changing the shapes of the bones and their articular surfaces. The case of which Fig. 8 is a skiagraph was a typical case of bad flat-foot of twenty years' duration. Fig. 17 is the photograph. The front part of the foot was abducted (see Fig. 8), the long internal arches reversed, the prolonged axis of the astragalus fell some distance internal to the head of the first metatarsal bone. The head of the astragalus could be felt dislocated in the sole; the anterior end of the long arch, the head of the first metatarsal, did not touch the ground; the bones in front of the mediotarsal joint were atrophied; "irritation osteophytes" are seen at the junction of the os calsis and cuboid; there was much new bone growth on the head of the astragalus; the last 1½ inches of the posterior tibial tendon was ¾ inch wide, doing the duty of a ligament, holding up the head of the astragalus. The mechanism was entirely changed.

### SYMPTOMS AND PHYSICAL SIGNS.

These are clear-cut. The long vertical internal arch is flattened; the long internal transverse arch is reversed. The foot is longer by just as much as the long internal vertical arch is longer than its projection upon the ground. The front part of the foot is abducted, and its outer edge rolled up. In the severer cases the scaphoid may be felt out of place, the bottom of the foot may be convex, and the posterior end of the heel raised. There is swelling just below the scaphoid, at the inner and outer sides of the ankle. Hallux valgus is often associated.

The feet usually perspire freely, are often hot, uncomfortably stiff and red, if the condition is acute. The gait is characteristically without elasticity and graceless.

Fig. 12.—Normal plantar flexion.

Fig. 13.—Normal dorsal flexion.

Fig. 14.—Normal adduction.

Fig. 15.—Normal abduction.

Figures 12, 13, 14 and 15 illustrate the normal ranges of motion in plantar flexion, dorsal flexion, adduction and abduction. In flat-foot these ranges of motion are always limited. Dorsal flexion in flat-foot is usually limited to ninety degrees; adduction is often completely lost. The foot is held in the abducted position by spasm of the peroneus longus (see Fig. 16). Atrophy of the weakened muscles is often a prominent sign. I have seen cases in which, without paralysis, the toes were practically useless, the patient hardly able to flex and extend them.

Fig. 16.

Fig. 17.

The whole foot may pain. The most prominent tender points are at the attachments of the tibial muscles, beneath the heel and beneath the external malleolus. Attacks of synovitis of the knee are frequently associated. There may be much backache from strain of the internal rotators of the thigh. Tenderness over the gluteal muscles may be present.

Figures 16, 17 and 21 illustrate the appearance of the foot in cases of average and greatest severity.

There are few if any cases of non-paralytic flat-foot that cannot be cured. The prognosis may be viewed from two standpoints:

1st, comfort; 2d, rectification of the anatomical defect. To obtain the first is absolutely essential, the latter most desirable. One cannot expect a flat-foot of mild or severe type to get well spontaneously any more than a broken-down bridge can raise itself without assistance. There are cases that do not have pain, unless the individual does a great deal of walking or standing, cases in which the ligamentous stretching, rotation and distortion of the bones has come to a standstill; cases in which there is no muscle spasm; cases in which coordination of the muscles has been partially established after having passed through the breaking-down, painful stage. Even these, however, suffer with knee pain, hip and backache, if much exercise is taken. Usually the patient has not even this comparative ease, the symptoms growing worse with time, leading to much discomfort and disability.

Often a patient is satisfied to become comfortable. Most often, lasting comfort is obtained only by rectifying the anatomical defect. Both should be the aim of the surgeon and both can be obtained. The time required to obtain this is of course variable. Often the cessation of discomfort is immediate and lasting from the application of foot plates. Usually it takes a little time to bring this about. It is a problem involving usually a few weeks of care in mild cases; sometimes months. A presentable foot can always be obtained, the degree of correction depending upon the degree of bone deformity present, tendon contractures, and the opportunity the patient may have for submitting to operative treatment. The cases resulting from acute or chronic arthritis and old sprains present no peculiar difficulties, and yield to treatment like the other cases.

When the defect is due to paralysis, the result depends upon what and how many muscles are affected and to what degree their lost function may be supplied by transplanting the tendons of good muscles into the paralyzed ones before applying the apparatus.

There is a large class of cases of mild degree presenting the usual symptoms with little or no spasm of the peroneus or extensor muscles. A case of this nature presents little limitation of the normal ranges of motion, but pain is produced when these motions are made to their normal limits. Such a type may be called "mobile flat-foot." The use of appropriate foot plates and

4

exercises for the weakened muscles soon reestablish foot poise and strength.

Often, however, the spasm of the muscles is so great that any effort to place the foot in the normal attitude is associated with unbearable pain. Under these circumstances, forcible correction is necessary, provided there be no osseous growth to prevent it. The patient is etherized; the foot is by manual force or the assistance of apparatus, brought to an adducted and plantar flexed position. Plaster is applied; the foot is allowed to remain in plaster from one to two weeks. Then the plaster is removed. After the plaster is removed the most careful attention is necessary to build up the muscles, to reestablish coordination so that foot poise is produced; exercises with the surgeon's hand resisting must be taken. Massage is advisable; the manner of walking must be carefully watched; plates must be applied and changed in shape as fast as the arch heightens and the front part of the foot becomes adducted. The shoes must fit properly. Infrequently one or more tendons are cut before it is possible to get the foot in a corrected attitude. The mechanical devise necessary is a spring arch so constructed that it holds the foot in the position it should have naturally. The plate must be absolutely comfortable, and must so support the weakened parts of the foot that relief from the flat-foot pains is obtained. The great problem in flat-foot is the shaping of these plates. If the plate presses too much here or there, the patient will have pain from the pressure. It must support the foot in just the way it needs assistance to relieve the flat-foot pains. The object is to accomplish the latter with the absence of the former.

Again many cases present callous places under the ball of the foot beneath one or more of the heads of the metatarsal bones. This adds an additional weight distribution problem. While all feet follow the general principles of weight distribution outlined above, each has its own individual deviations. Each presents its own problem to be solved. Hence, there is as much difference in the individual necessary forms of support as there is difference in the facial appearance of people, who look alike.

Fig. 18 illustrates the type of plate ordinarily used. The plates are made of tempered spring steel, gauges sixteen to twenty, depending upon the weight of the patient, and the necessary breadth of the splint.

Fig. 18.—A type of splint.

To make a plate a cast of the foot is necessary. The cast is taken of the patient's foot in the corrected position, as near the position illustrated in Fig. 1 as possible. The cast is then carved in such a way that the carved cast represents the way the bones of the tarsus and metatarsus are to be supported. The area the splint is to fit beneath and support is then marked out upon the under surface of the cast. A mechanic makes the splint to fit this area. The splint is then inclosed between an upper and lower piece of leather. The completed splint then slips in the shoe like an insole. The plate will go in any shoe that the patient can wear with comfort.

The cases reported are typical mild and severe ones. These three are selected to show some of the procedures in treatment.

CASE 1.—Fig. 17 is a photograph of one of these cases. Fig. 8 is the skiagraph. The flat-foot had been present twenty years. The condition followed an attack of acute anterior poliomyelitis. The posterior tibial was paralyzed, the flexor longus pollicis, anterior tibial and soles weakened. Flat-foot followed. The internal arch was destroyed so that its anterior end, the head of the great metatarsal, did not touch the ground when the patient walked. The pain in the ankle and across the instep was great. The head of the astragalus could be felt in the sole. Foot plates were applied without relief. Other apparatus was tried without relief. Fig. 8 explains the difficulty. Compare this with the photograph of the normal foot (Fig. 5). Note in Fig. 8 the enlargement of the head of the astragalus. It was necessary to chisel out most of the head of the astragalus before the foot could be straightened. The head of the great metatarsus and the distal end of the first phalangeal bone were also resected before the proper relation of the bones of the internal arch could be reestablished. This patient is still being treated, so the result is not shown. This case shows the great pain, disability that must come, the failure of apparatus to help those cases in which there is body obstruction to the mechanical restorative effort, and the necessity of an extensive bone operation where the foot skeleton is greatly deformed.

Case 2, Fig. 19.

Case 2, Fig. 20.

Case 2.—Fig. 19 illustrates some of the steps taken in mobile flat-foot. The posture of flat-foot is seen, the cast taken of the foot in the proper position, the cast carved with splint marked out upon it and the alteration made in the heel of the shoe. Fig. 20 shows the foot anatomically and functionally restored after several weeks of exercises and wearing a plate. This patient had pain in the ankle, especially at the attachment of the posterior tibial muscle, and became very tired after walking short distances. The discomfort has passed away after treatment.

Case 3, Fig. 21.—Photograph of foot before operation

Case 3, Fig. 22.—Skiagraph of foot before operation.

Case 3.—The child was three and one half years old at the time she was stricken with paralysis, a typical attack of anterior poliomyelitis. For a time both legs were paralyzed. She is now nine years old. All the muscles in both legs recovered, some partially, some completely, except the tibialis anticus, tibialis posticus and flexor longus pollicis of the right leg. These muscles remained completely paralyzed. The result of this paralysis was the deformity shown in Fig. 21. The child walked with the foot turned out about sixty degrees, and with bad rolling gait. The internal arch was absolutely destroyed, the internal malleolus almost touching the ground. The tendo achillis was much shortened, so that it was impossible to dorsally flex the foot without first abducting it. The peroneus longus and brevis feeling no opposition, held the foot strongly in the abducted position.

Case 3, Fig. 23.—Photograph of foot after operation.

The shoe in Fig. 25 shows the appearance presented by the clothed foot before operation. See from the posture of the shoe how the outer edge of the shoe rolled up and to what extent the internal arch was destroyed.

Fig. 5 shows the skeleton (skiagraph) of a foot functionally normal. Notice that the prolongation of the axis of the astragalus, direction of the arrow, passes very close to the head of the great metatarsal. In normal feet sometimes the axis of the astragalus passes through the head of the great metatarsal, but usually just a little to the inner side. Notice also the contour of the inner and outer borders of the foot skeleton and the size of the inner row of bones.

Fig. 22 is a skiagraph of the child's foot taken before operation. The striking features of the foot skeleton are at once evident. The prolongation of the axis of the astragalus falls some distance internal to the head of the great metatarsal. The head of the astraga-

Case 3, Fig. 24.—Skiagraph of foot after operation.*

lus is not rounded as it should be. All the bones of the foot are undeveloped from not having been used as much as they should; particularly is this true of the scaphoid, internal cuneiform and great metatarsal. The astragalus is displaced forwards and inwards, the outer half of the mediotarsal joint between cuboid and os calcis being behind the inner half formed by the astragalo-scaphoid

*In making the exposure of the foot in Fig. 24 the back of the X-ray plate was inadvertently placed next the foot instead of the film side. This reversed the image and makes the foot in the illustration appear to be the left instead of the right foot as it really was, as appears in Fig. 22.

articulation. The front part of the foot is markedly abducted. The whole foot is smaller and shorter than the other. The skiagraph shows in addition to the abnormal position of the bones, the lack of development from disuse and the slight skeleton deformity already developed, which later would have been marked and fixed from the changed pressure and weight conditions and mechanical action of the foot.

The child was operated upon last April. The tendon of the peroneus longus was detached at the outer edge of the foot, split

Case 3, Fig. 25.—Shoe worn before operation. Cast of foot taken after operation, carved and splint marked out.

and transplanted one half into the tendon of the paralyzed tibialis posticus and one half into the flexor longus pollicis behind and a little above the internal malleolus. The tendo achillis was lengthened, the long extensor tendons of the second, third and fourth toes were transplanted into anterior tibial. In this way the foot was restored to the proper shape.

Fig. 23 shows the naked foot after operation. The correction of the deformity is obvious. Great care was necessary by massage exercises, the hand resisting, to develop a sense of responsibility in the muscles for the correct performance of their new work and to drill the child out of the former habits of walking and standing, for had the foot not been used rightly after operation while it was weak, it would have partially relapsed.

Fig. 26 shows the child standing, ten months after the operation. Contrast the right foot with the posture of the shoe in Fig. 25. Within the right shoe in Fig. 26 is a small 20-gauge steel splint which fits beneath that part of the foot marked out on the cast in Fig. 25. The cast was taken after the operation, and then carved beneath by hand in such a way that the splint moulded on

Case 3, Fig. 26.—Standing after operation.

this cast, when fitted beneath the foot, would supplement the muscular and ligamentous support to the internal arch. The splint is comfortable, supports the foot, and is not visible. The deformity is corrected, the child's locomotion is vastly improved and will improve more as time goes on, for the muscles left in the weak leg will become stronger, since they now work with a mechanical advantage. The foot will now develop properly. Had this foot been allowed to remain as it was before the operation, several years later the skeleton would have presented the deformed structure shown in Fig. 8.

## Figures and Tables

**Fig. 1. f1:**
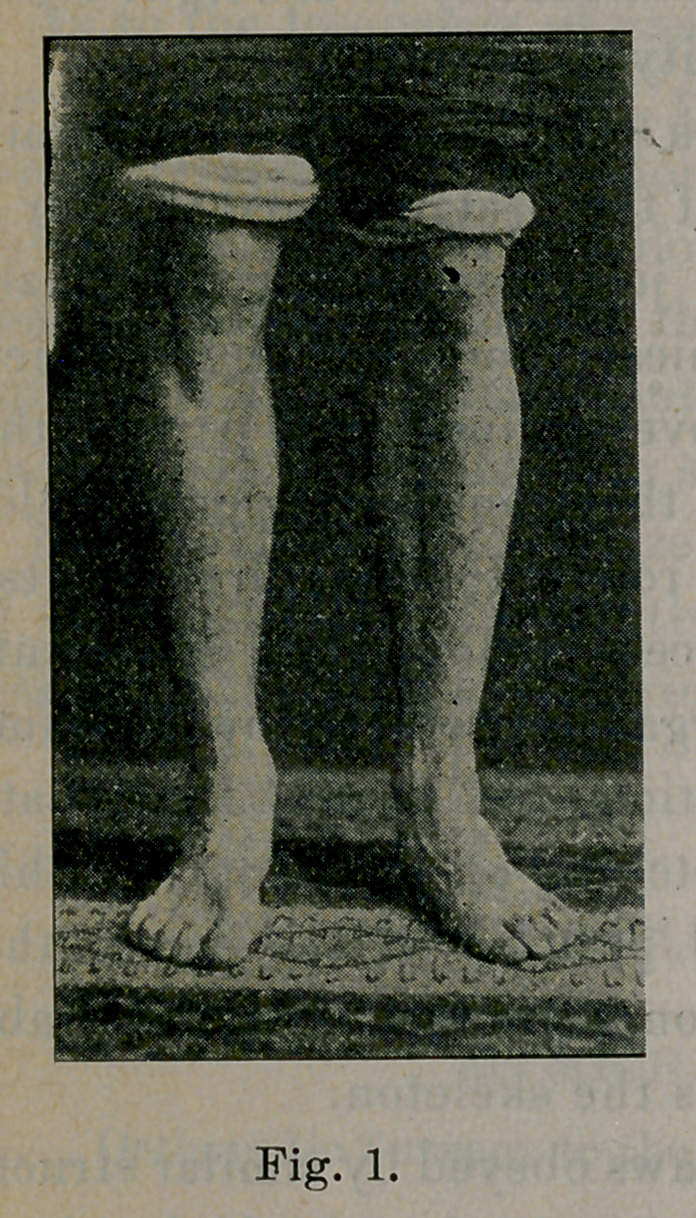


**Fig. 2. f2:**
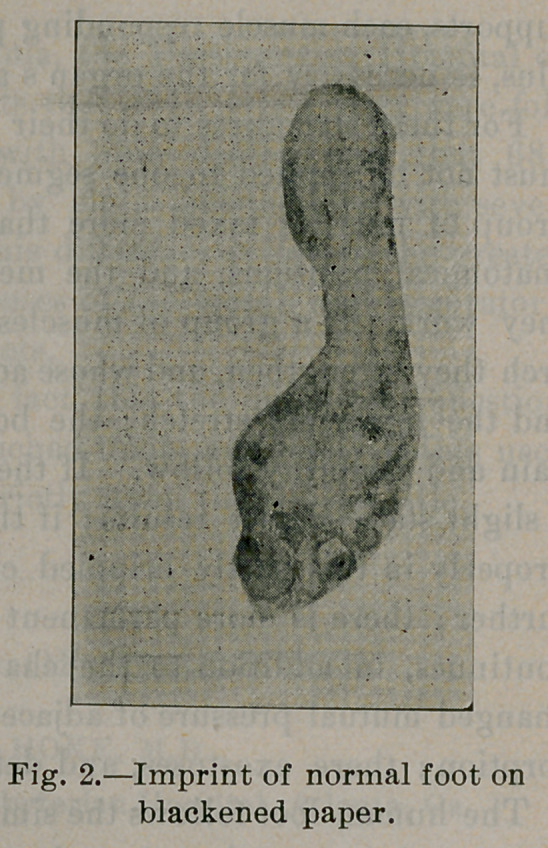


**Fig. 3. f3:**
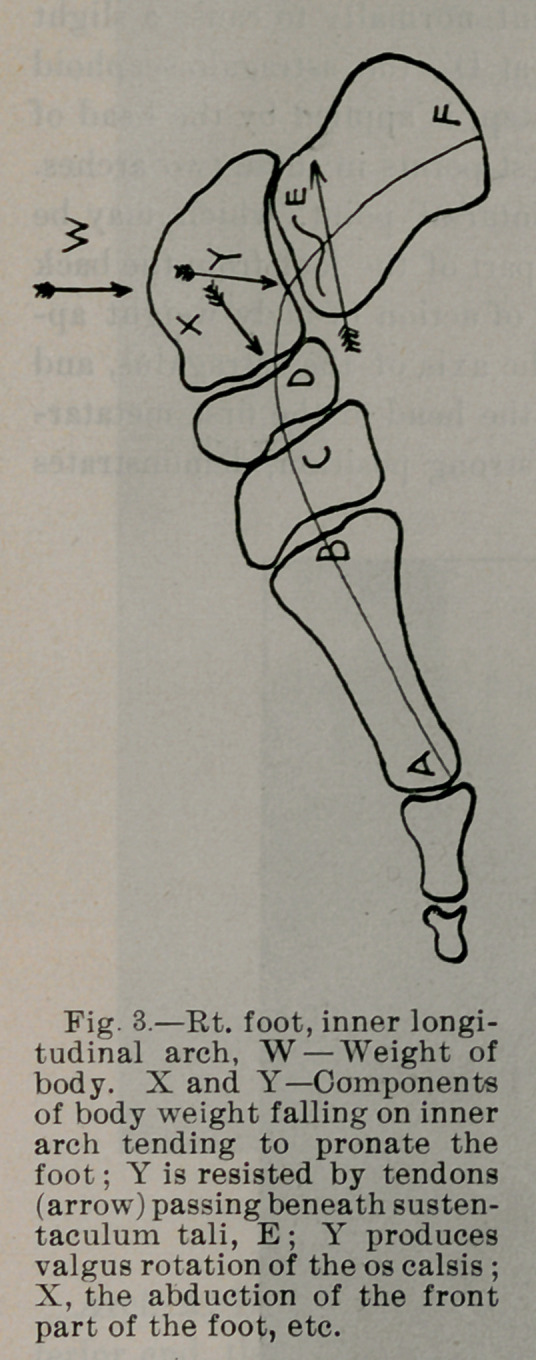


**Fig. 4. f4:**
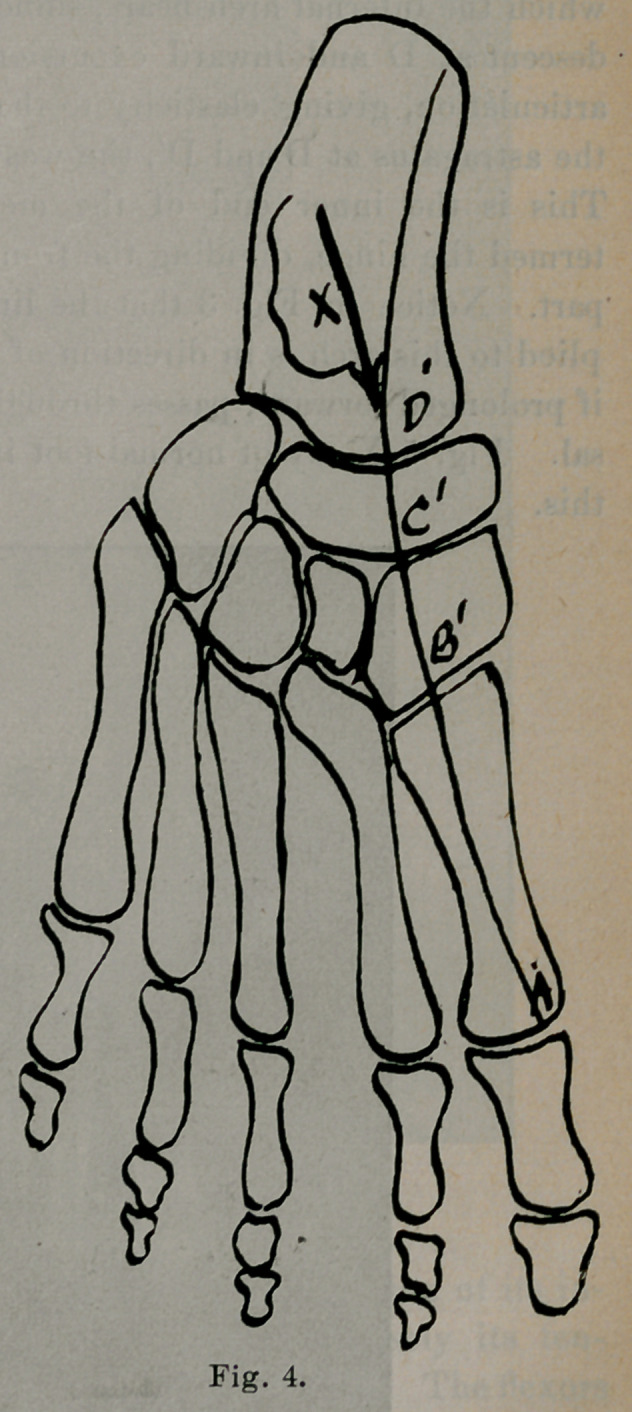


**Fig. 5. f5:**
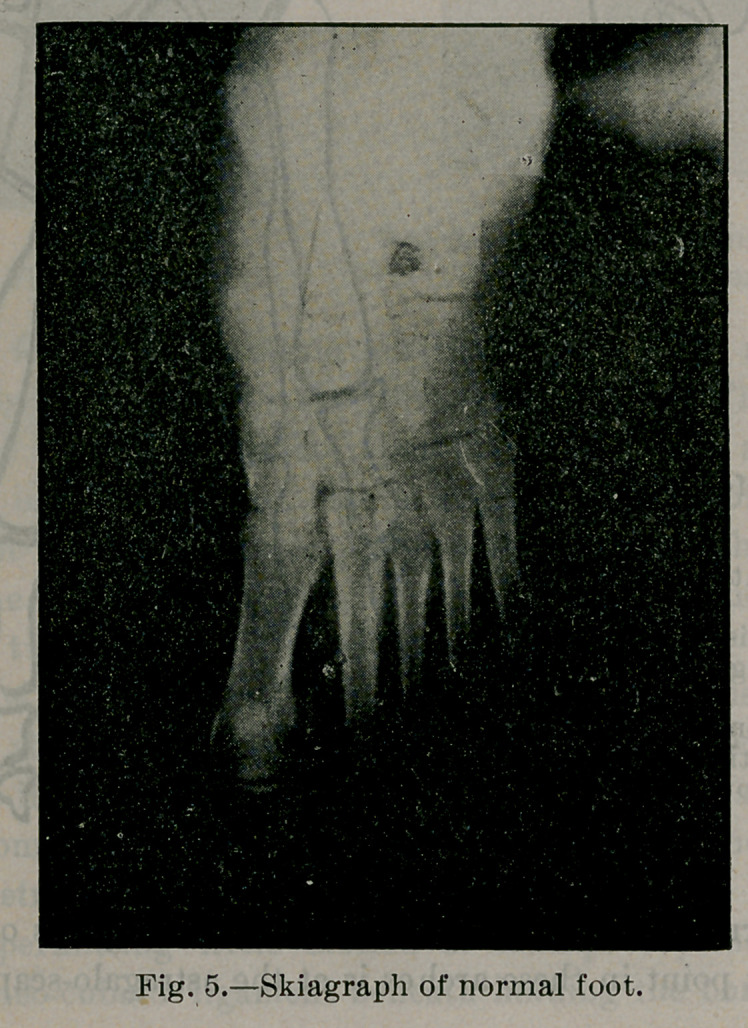


**Fig. 6. f6:**
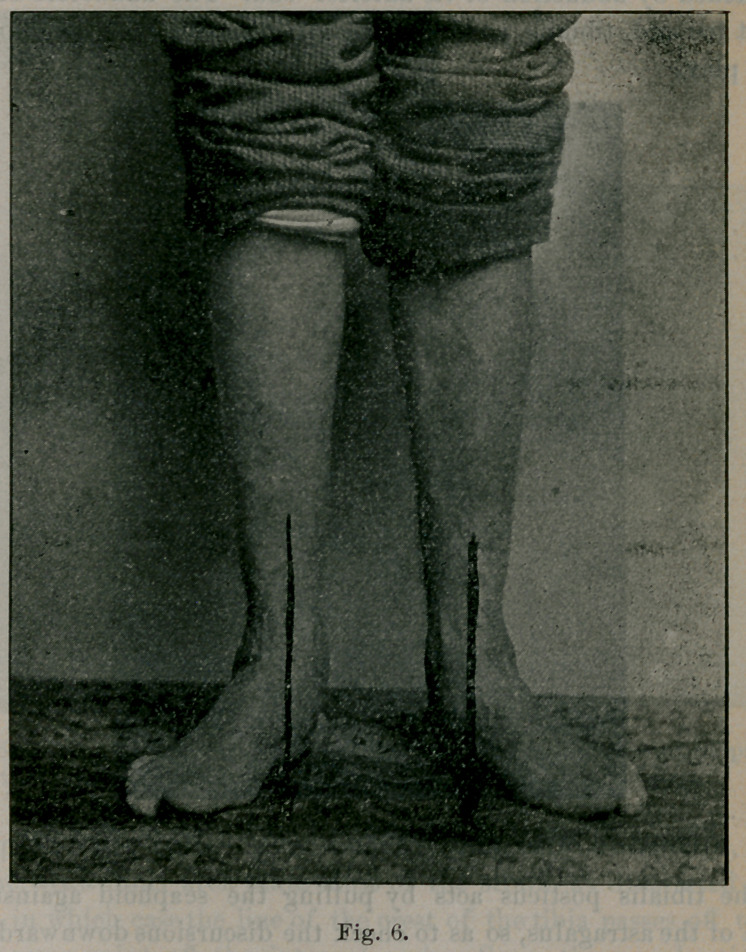


**Fig. 7. f7:**
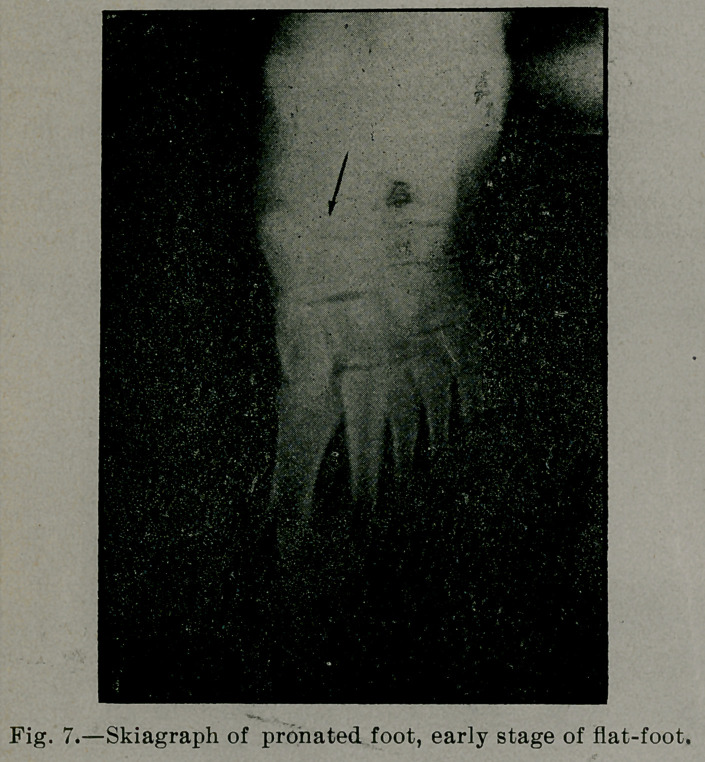


**Fig. 8. f8:**
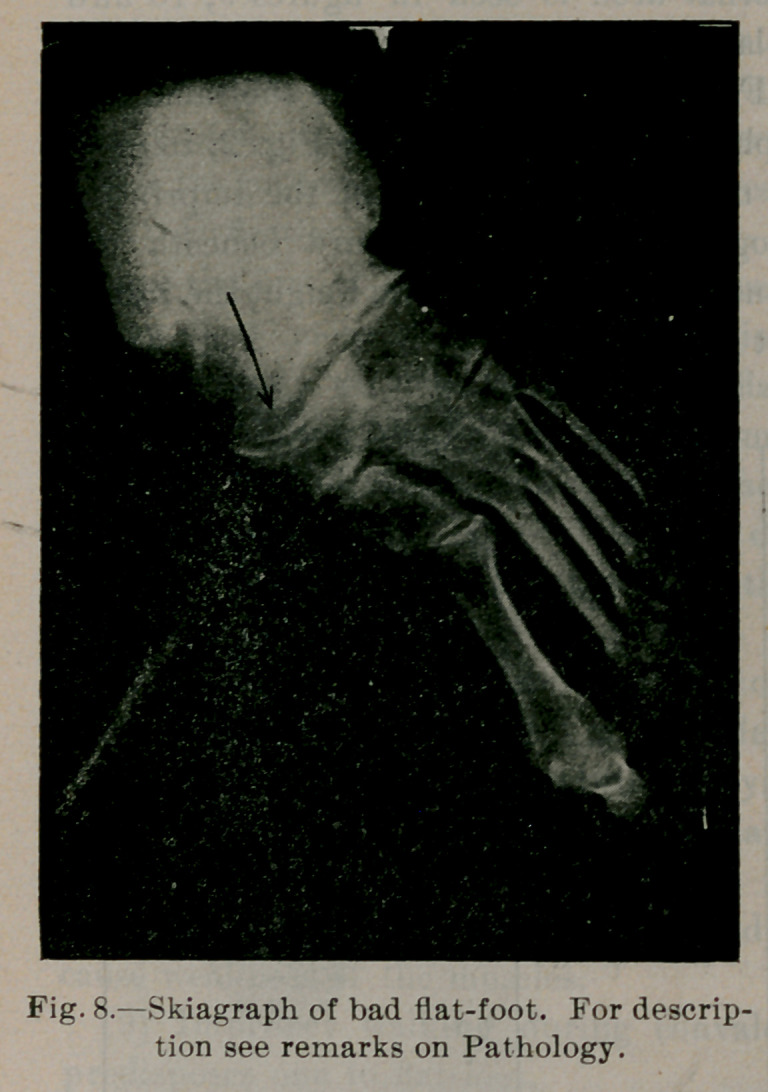


**Fig. 9. f9:**
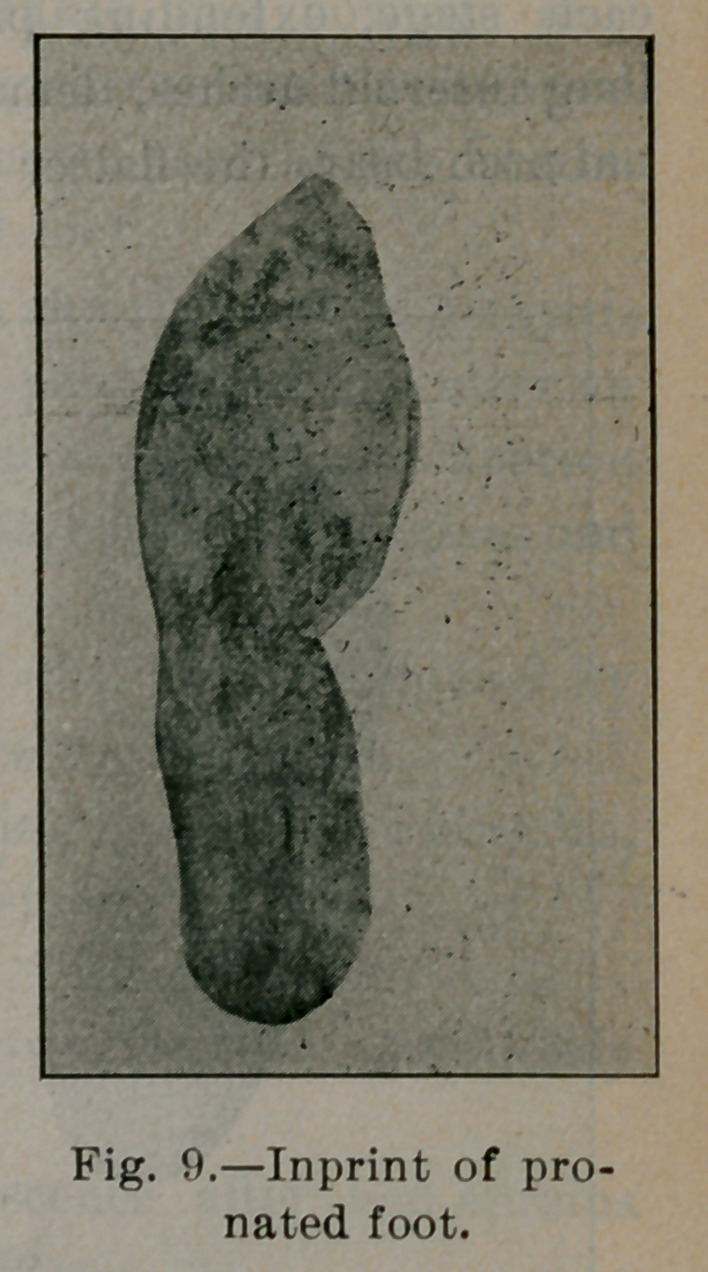


**Fig. 10. f10:**
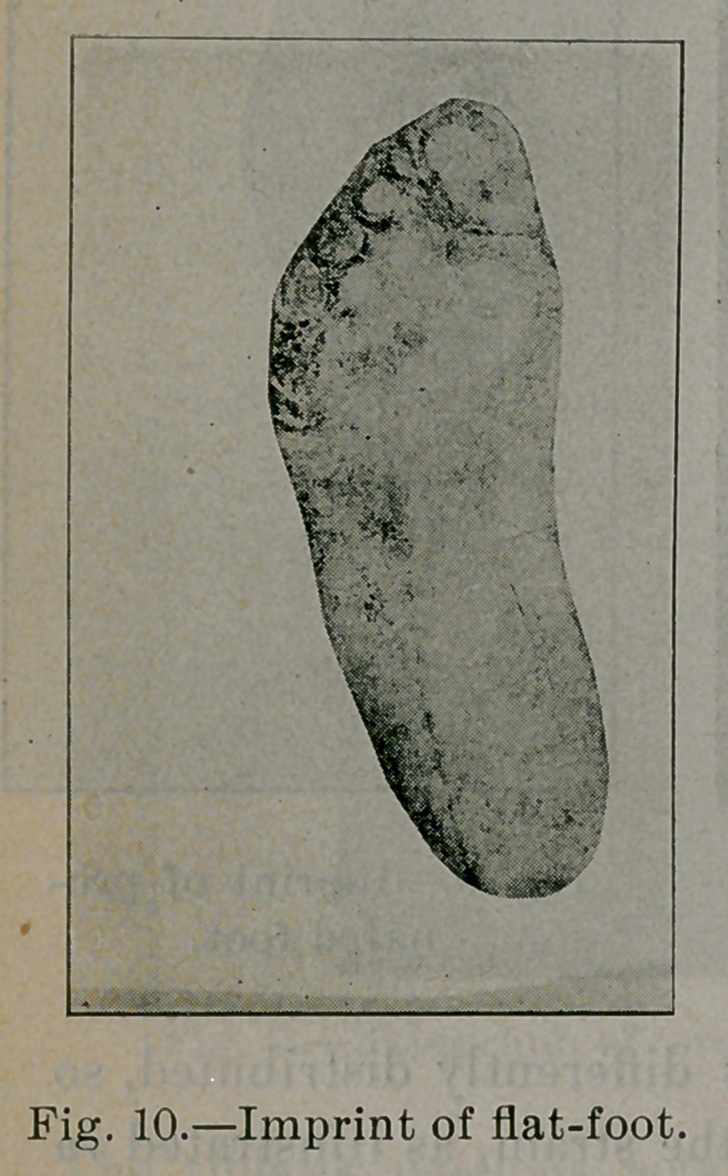


**Fig. 11. f11:**
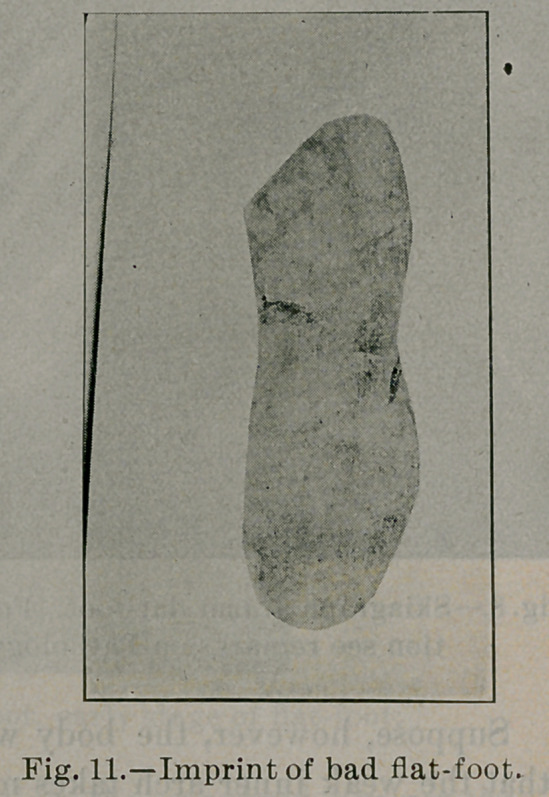


**Fig. 12. f12:**
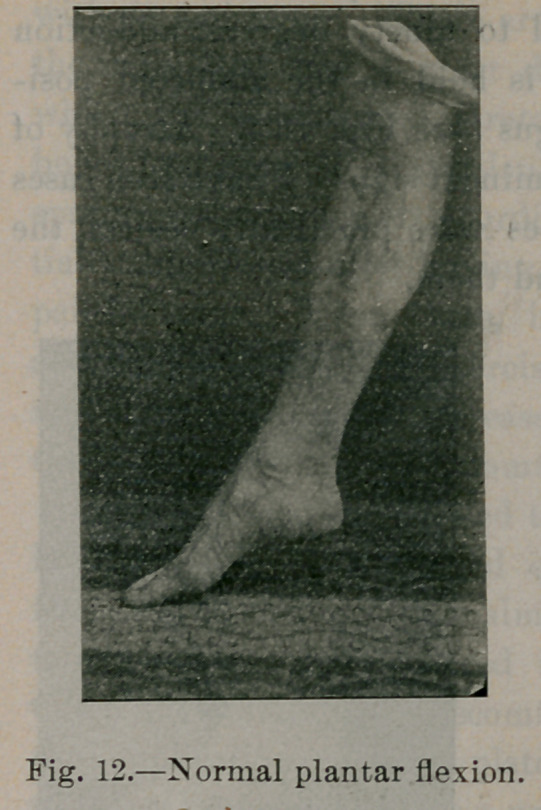


**Fig. 13. f13:**
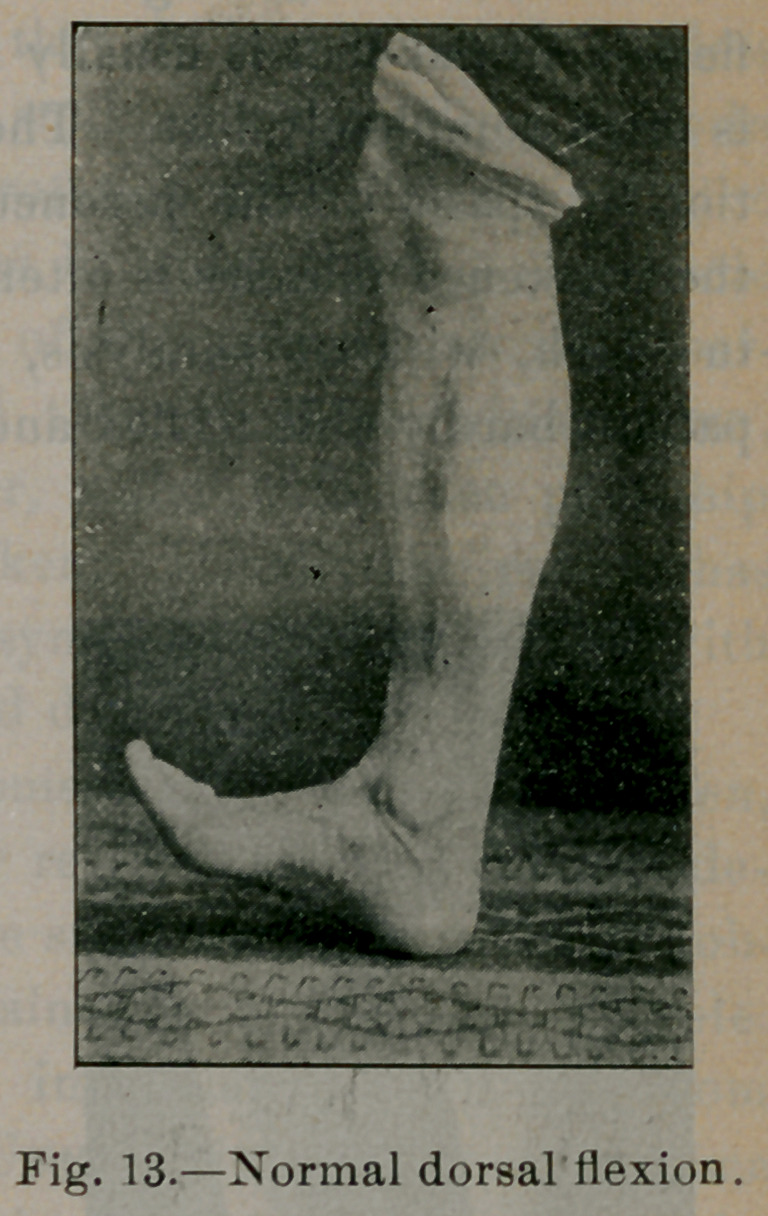


**Fig. 14. f14:**
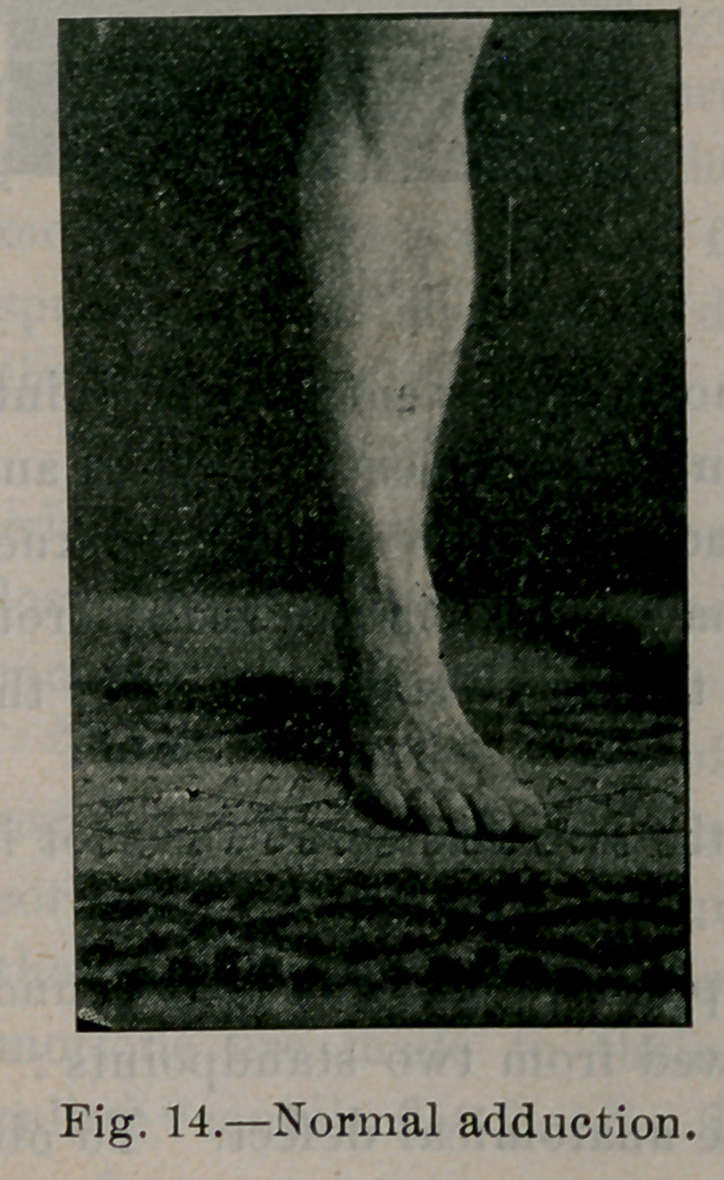


**Fig. 15. f15:**
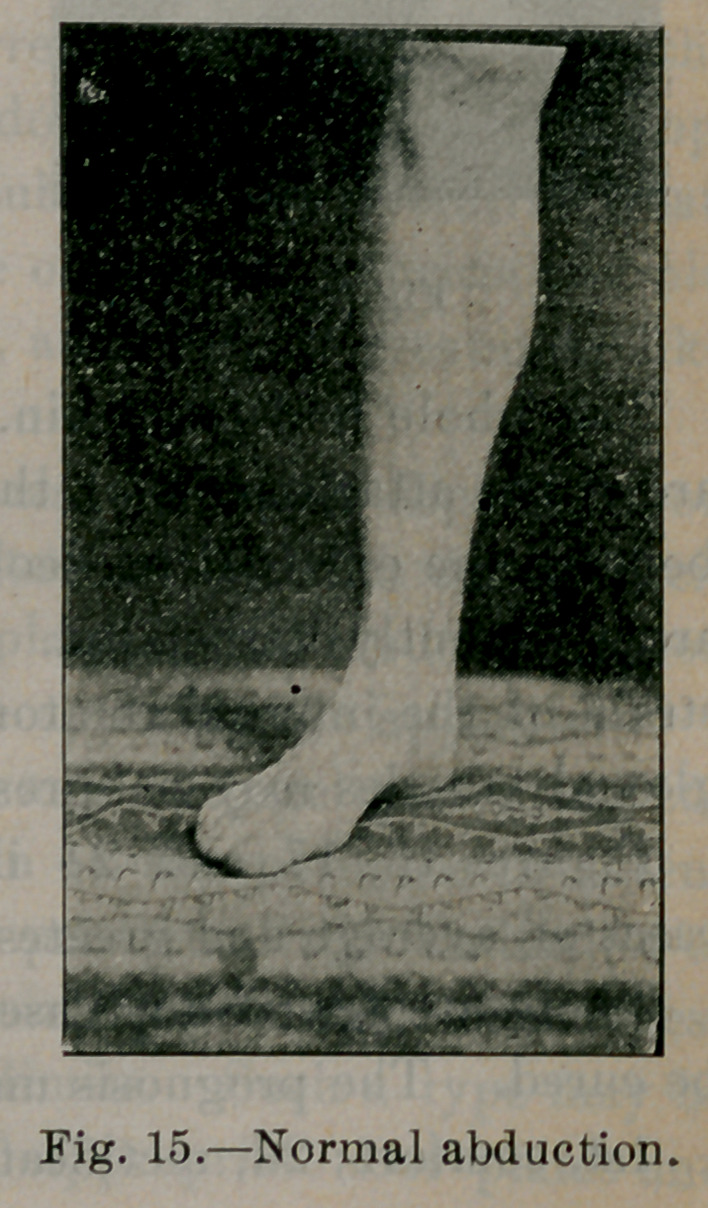


**Fig. 16. f16:**
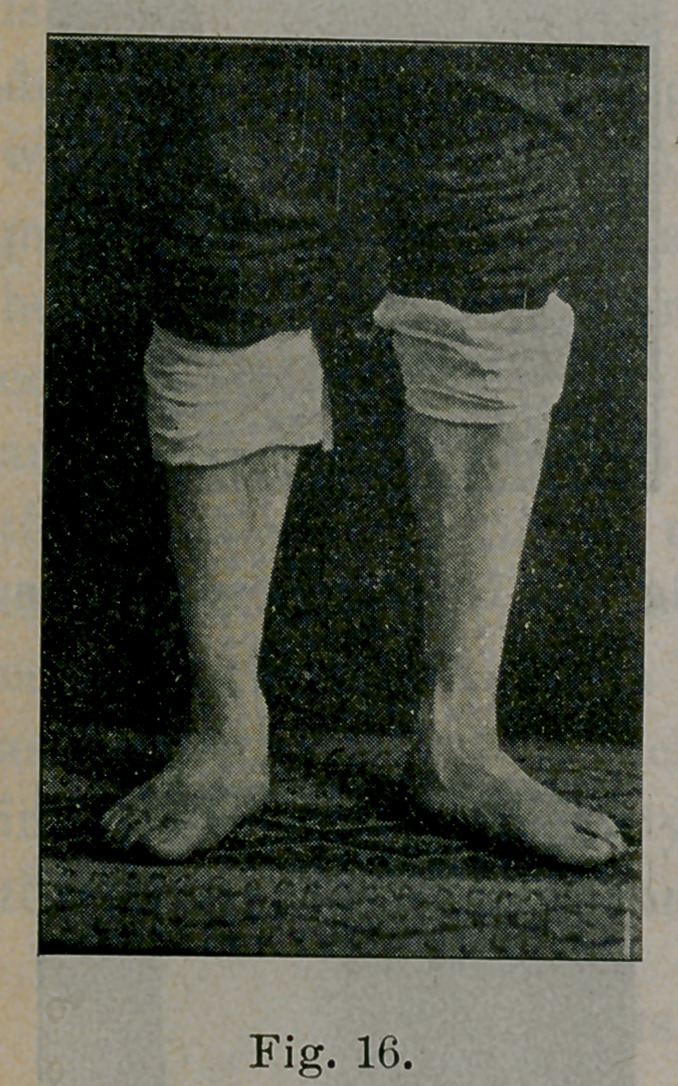


**Fig. 17. f17:**
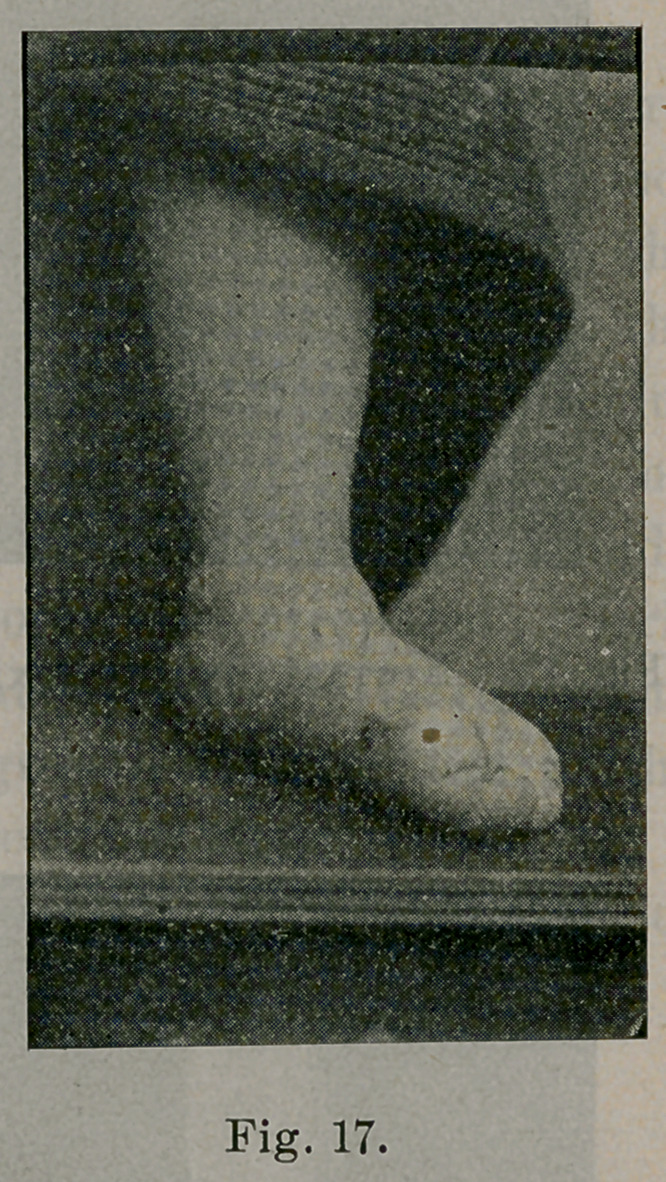


**Fig. 18. f18:**
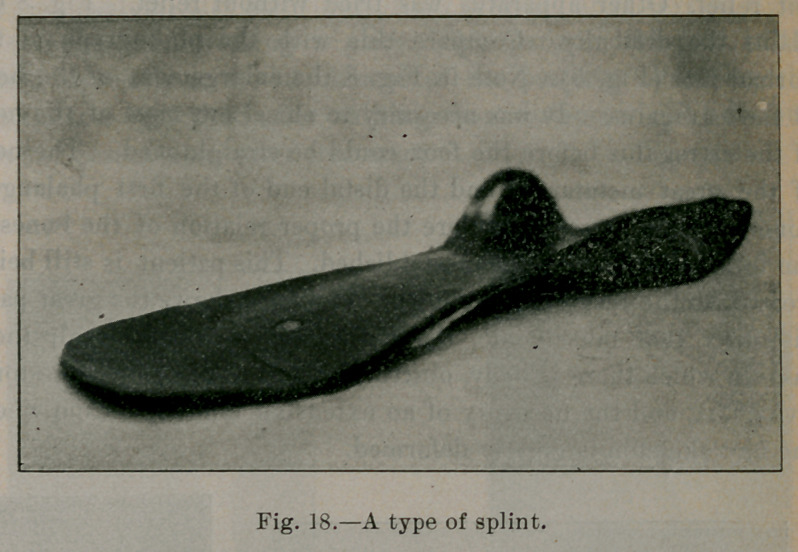


**Case 2, Fig. 19. f19:**
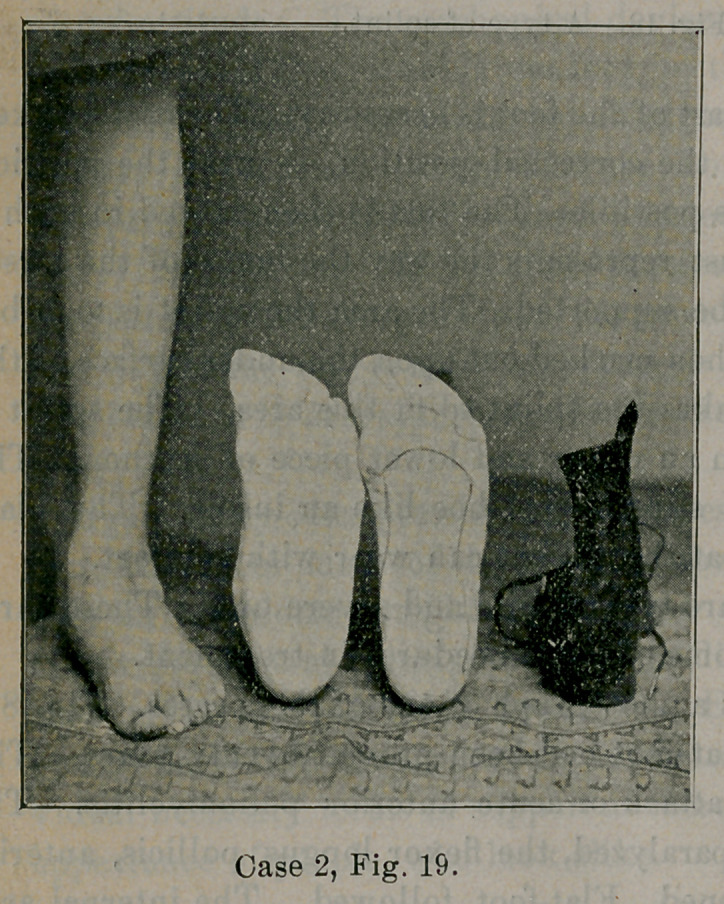


**Case 2, Fig. 20. f20:**
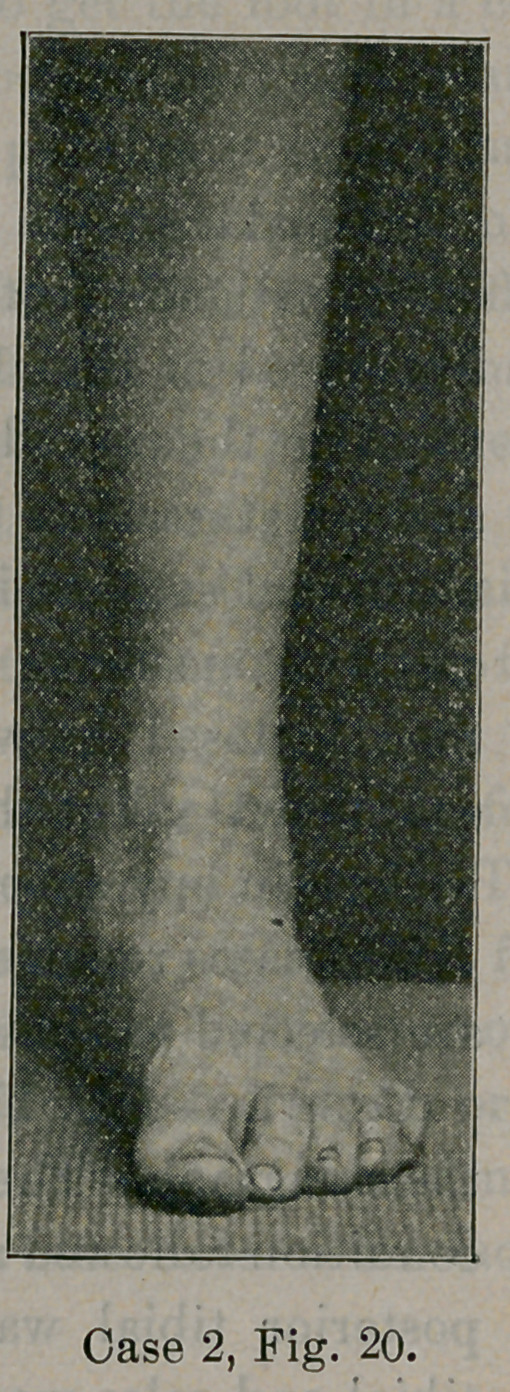


**Case 3, Fig. 21. f21:**
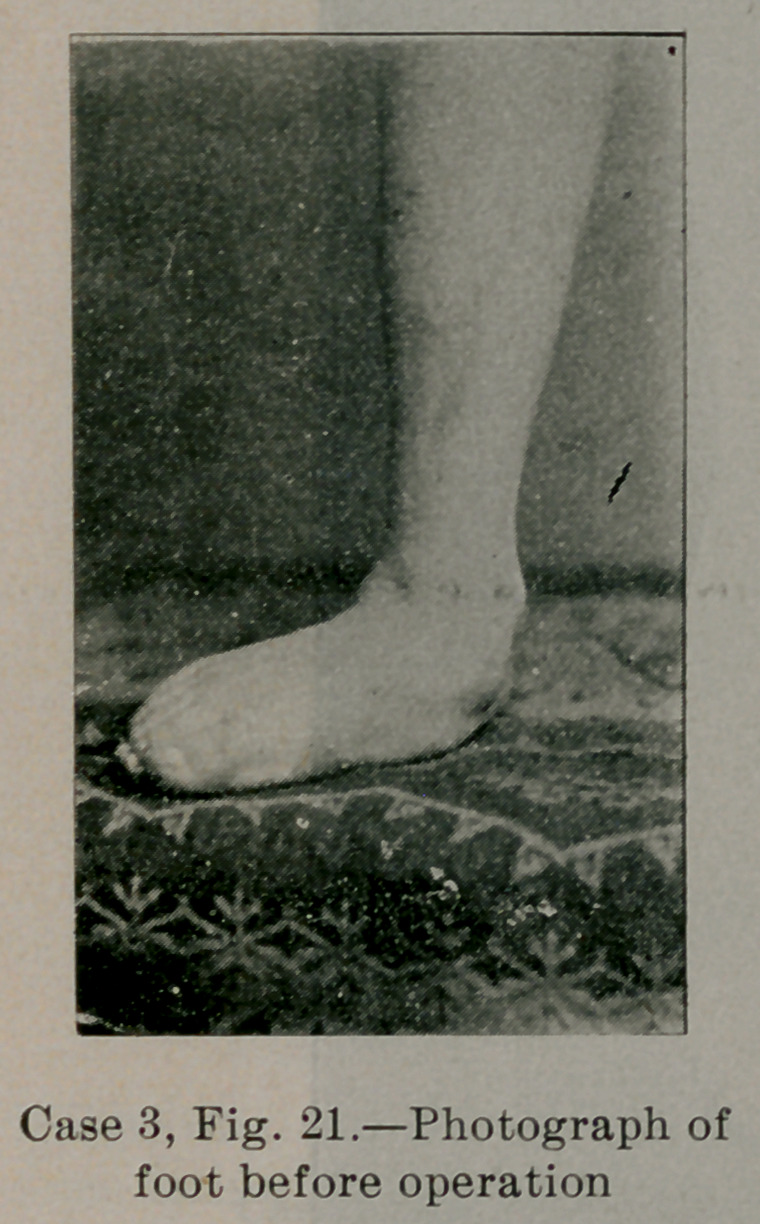


**Case 3, Fig. 22. f22:**
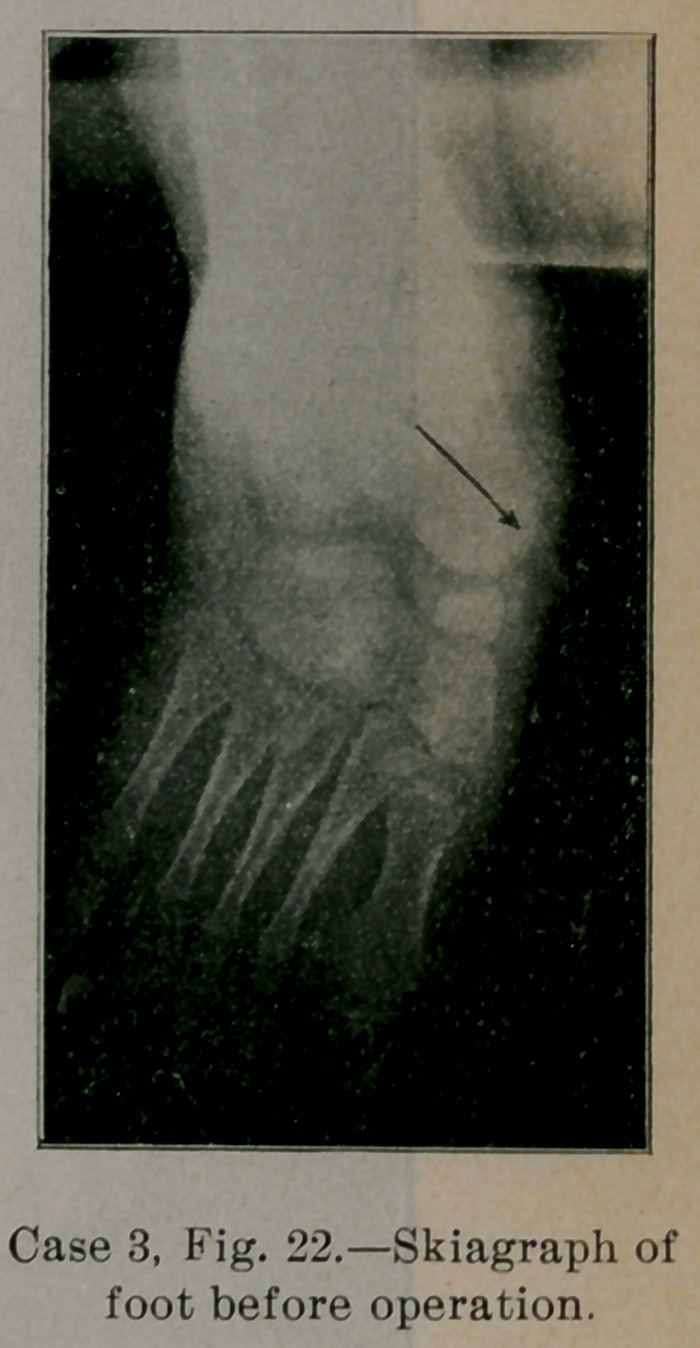


**Case 3, Fig. 23. f23:**
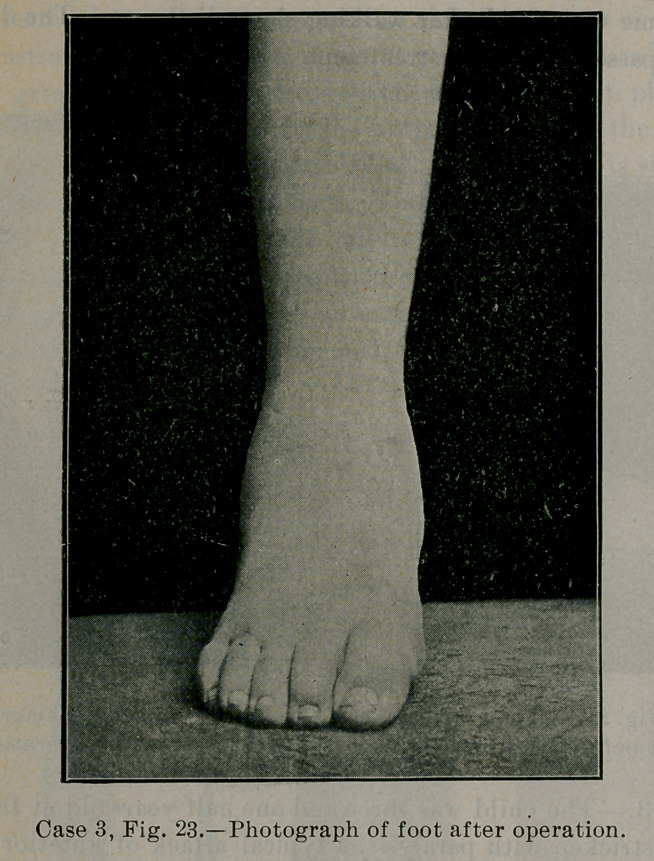


**Case 3, Fig. 24. f24:**
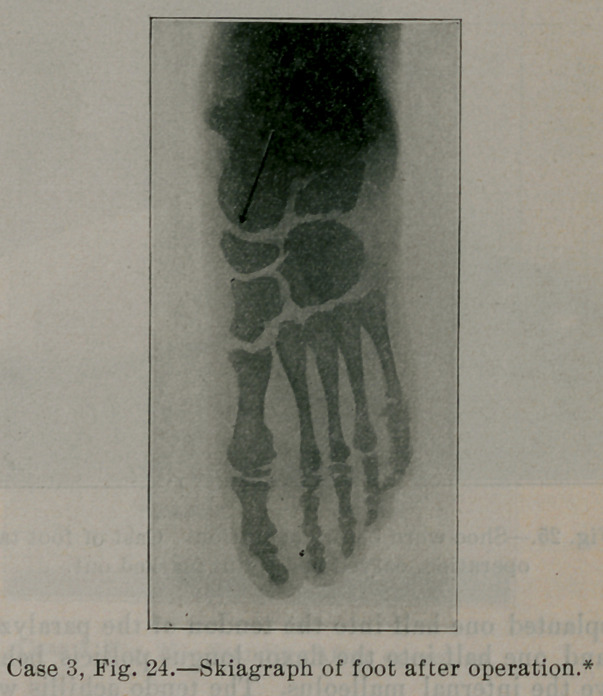


**Case 3, Fig. 25. f25:**
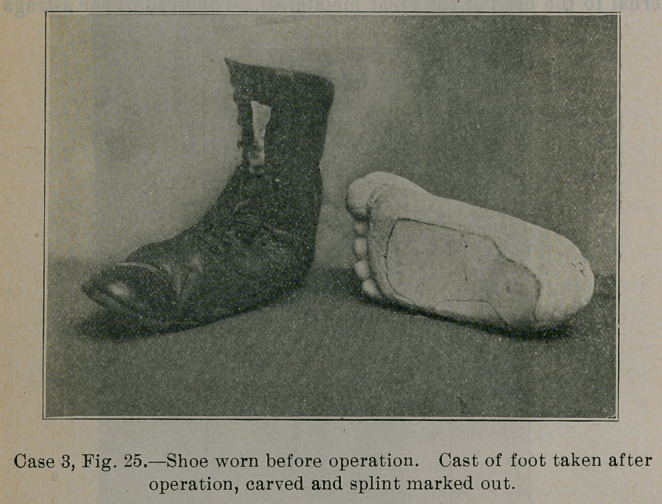


**Case 3, Fig. 26. f26:**